# Rare case of spontaneous predominantly posterior infarction of the conus medullaris: MRI clears the dilemma

**DOI:** 10.1007/s00415-025-13165-2

**Published:** 2025-05-20

**Authors:** Robert Forbrig, Kasturi Rangan, Andreas Schindler

**Affiliations:** 1 Institute of Neuroradiology, University Hospital, Ludwig-Maximilians-University Munich, Marchioninistr. 15, 81377 Munich, Germany; 2 Department of Radiology and Nuclear Medicine, Medanta Hospital, Lucknow, India

**Fig. 1:**
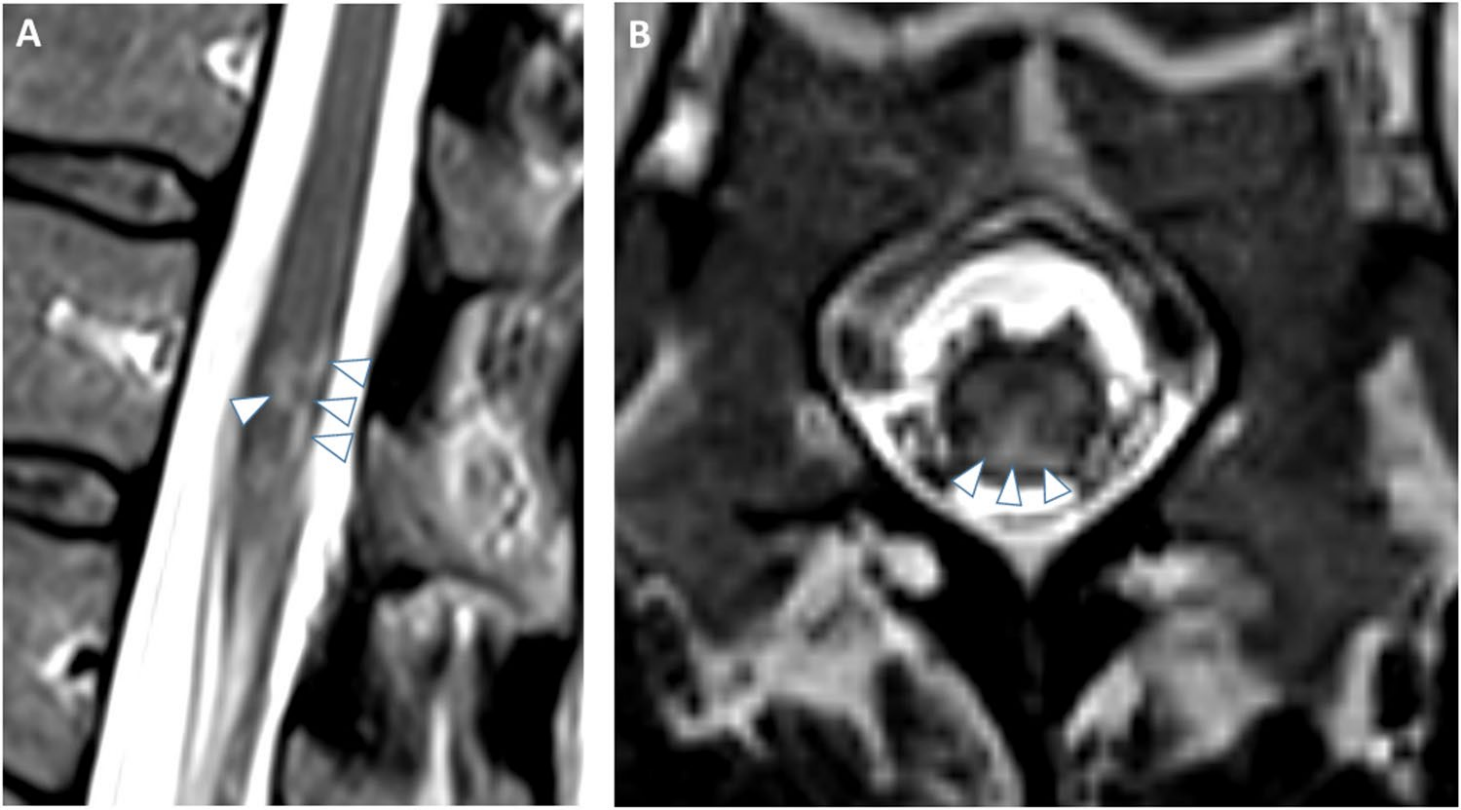
T2-weighted imaging in sagittal (**A**) and transversal (**B**) planes reveal bilateral hyperintensities (arrowheads) affecting the medullary gray matter at the conus level, predominantly affecting the posterior portion

**Fig. 2:**
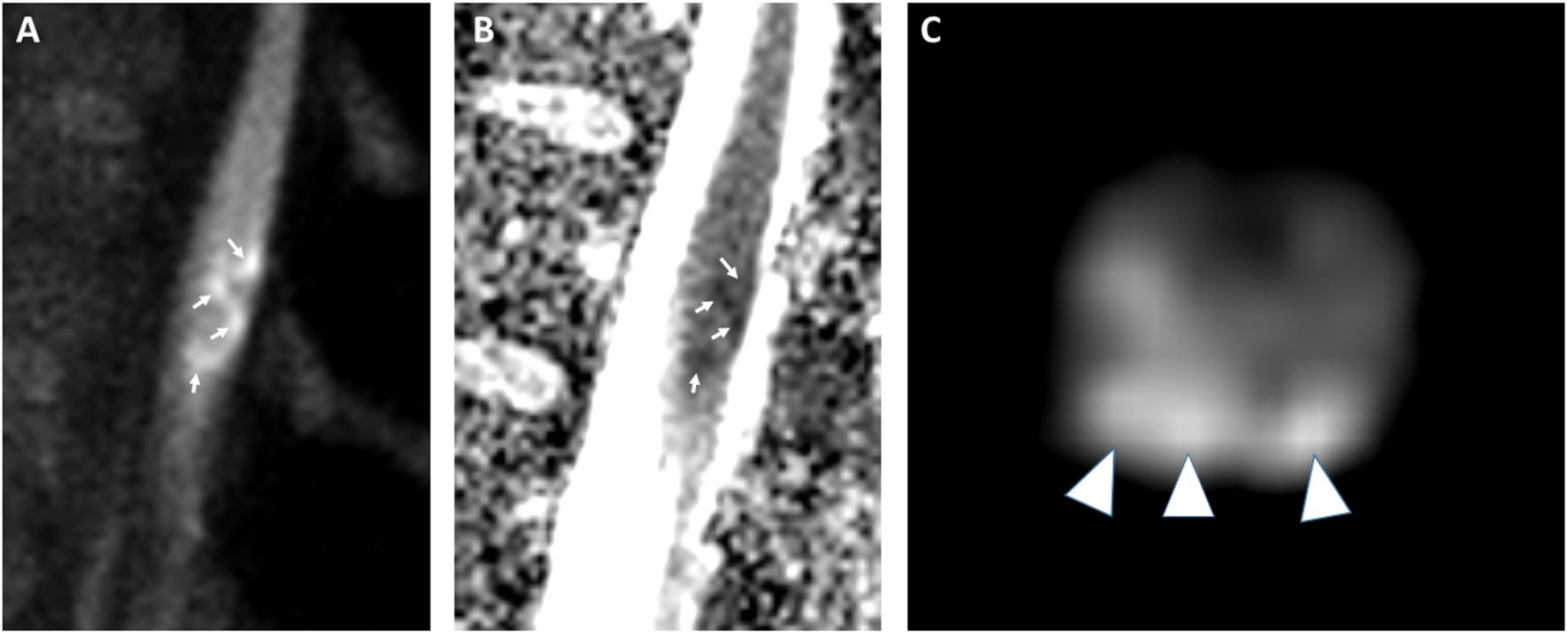
Sagittal diffusion weighted imaging (DWI) shows diffusion restrictions (arrows in **A**, b600; arrows in **B**, apparent diffusion coefficient) matching the T2-hyperintensities and consistent with acute conus medullaris infarction. Transversal DWI confirms the predominantly posterior involvement (arrowheads in **C**, b600)

Dear Sirs,

A 45-year-old female with an unremarkable medical history presented with acute lumbar pain, saddle anesthesia, loss of bladder and bowel control, and progressive sensory deficits in the lower limbs developing over several hours. Motor function in the legs remained preserved. Initial laboratory tests and cerebrospinal fluid (CSF) analysis, as well as lumbar MRI with T1- and T2-weighted sequences 6 h after symptom onset, were normal. Due to the clinical suspicion of (autoimmune) myelitis, high-dose corticosteroid therapy was initiated; however, symptoms persisted. A follow-up MRI performed 30 h after symptom onset revealed new medullary T2-hyperintensities, predominantly posterior, without contrast enhancement, bilaterally involving the gray matter of the conus medullaris (Fig. 1). Additional diffusion-weighted imaging (DWI) demonstrated corresponding diffusion restriction, consistent with infarction of the conus medullaris (Fig. 2).

Spinal cord infarction accounts for less than 1% of all strokes and is therefore a rare clinical entity [1]. Isolated infarction of the conus medullaris is exceedingly uncommon due to the region’s extensive microvascular anastomoses between the singular anterior and two posterior spinal arteries, referred to as the “arterial basket” [2]. Conus infarctions may affect the anterior aspect of the cord or, more rarely as in our case, the posterior portion. Regarding the etiology, spinal cord infarction may arise spontaneously or in association with medical procedures, particularly aortic surgery [3]. Spontaneous etiologies include aortic dissection or aneurysm, atherosclerosis, embolic events (e.g., fibrocartilaginous embolism), cervical artery injury, and rare conditions such as lupus, sickle cell disease, or vascular malformations [3, 4]. Periprocedural causes account for up to 45% of cases and include aortic cross-clamping, spinal angiographic procedures, epidural injections, and lumbar or orthopedic surgery. Risk factors include prolonged ischemia time, existing vascular disease, and embolic complications [3]. Despite extensive evaluation, no clear cause is identified in a substantial proportion of cases.

MRI is the modality of choice for diagnosing spinal cord ischemia, though initial scans may appear normal in one out of four cases [1, 3, 4]. Imaging protocols should include sagittal and axial T1- and T2-weighted sequences, post-contrast imaging, and DWI, which is highly sensitive for detecting ischemia through characteristic diffusion restriction. However, in contrast to cerebral infarction, DWI may be negative in spinal cord ischemia within 24 h after symptom onset [3]. Intravenous administration of gadolinium can help differentiate ischemic from non-vascular etiologies, with enhancement typically appearing only in the subacute phase of spinal infarction. Characteristic MRI signs include the “pencil-like” T2 hyperintensity on sagittal images, “owl’s eye” sign of anterior horn involvement, and “positive anterior cauda” sign, among others [3, 4]. Although rare, adjacent vertebral body or disc infarction on MRI is highly specific for a vascular cause.

The acute onset of conus medullaris syndrome, characterized by sensory deficits, autonomic dysfunction, and preserved motor strength, warrants consideration of several differential diagnoses. Inflammatory etiologies such as transverse myelitis or neuromyelitis optica spectrum disorder (NMOSD) are important differentials, particularly in the early stages when MRI findings may be inconspicuous. Infectious causes, including viral (e.g., varicella-zoster virus, HIV) and bacterial agents (e.g., Treponema pallidum, Borrelia), can present with similar symptoms, often accompanied by systemic signs or abnormal CSF parameters [3]. Structural lesions such as epidural hematomas, neoplasms, or large herniated discs should be excluded via high-resolution spinal imaging [3, 4]. Vascular anomalies – including spinal arteriovenous malformations and dural arteriovenous fistulas – can also present with conus symptoms and are best identified through contrast-enhanced MR-angiography and conventional angiography [5]. Furthermore, metabolic conditions such as vitamin B12 deficiency may mimic conus syndromes, though typically with a more chronic progression [3].

In summary, we report a rare case of spontaneous conus medullaris infarction predominantly affecting the posterior portion, an uncommon localization. Initial imaging was unremarkable, and the diagnosis was only confirmed after 30 h through diffusion-weighted MRI. This case underscores the importance of high clinical suspicion, timely re-evaluation, and prompt treatment, particularly during the hyperacute phase of spinal ischemia when conventional imaging and laboratory findings may still be inconclusive.
